# Postpartum Care Program in Japan

**DOI:** 10.3389/fgwh.2024.1333758

**Published:** 2024-03-08

**Authors:** Shunji Suzuki

**Affiliations:** Department of Obstetrics and Gynecology, Nippon Medical School, Tokyo, Japan

**Keywords:** Postpartum Care Program, Japanese mother, midwives, mental healthcare, Postpartum Maternity Checkup Program

## Introduction

The Postpartum Care Program was implemented in Japan in 2024 to support healthy childcare for mothers and their families by promoting postpartum physical recovery and mental rest for mothers and children by fostering self-care skills in mothers themselves (1). The medical and local government staff have to be proactive because mothers who are at risk of postpartum depression tend not to seek support by themselves (2). In Japan, therefore, the mental status of mothers is examined along with their physical recovery and lactation status at 2 weeks and/or 1 month after delivery at obstetric institutes (Postpartum Maternity Checkup Program). The results are then reported to the local government, enabling the Postpartum Care Program to provide support to mothers who require mental support (3).

## Current status of the Postpartum Care Program project in Japan

Since the publication of the “Guidelines for Prenatal and Postpartum Support Services and Postpartum Care Program” in 2017, it has been utilized as an implementation outline for postpartum care in each municipality (1). The Postpartum Care Program became a legal requirement in December 2019 and was imposed as a duty of effort on the part of local governments from April 2021. In May 2020, the Fourth National Outline of Measures for Society with Declining Birthrate in Japan stated that the program should be implemented nationwide by March 2025, and the guideline mentioned above was revised in August 2020. The main cost of the Postpartum Care Program will be subsidized half by the national government and half by local governments, and the co-payment of the user is determined by local conditions and the income of the user. The service can be provided in three types: (a) short-term residential care (overnight stay or short stay), (b) daycare, and (c) home visitational care (outreach), which are provided at obstetric hospitals, obstetric private clinics, midwifery centers, and other institutes according to the standards set by the Ministry of Health, Labor and Welfare of Japan (1). The targets of the subsidy program are mothers and children with postpartum physical and/or mental problems and/or anxiety about childcare and those considered to need financial/social support.

On the other hand, the Postpartum Care Program has been subsidized for mothers and children who have a certificate of residence (pay resident tax) in the municipality (3, 4). Because the scale of the local program differs among municipalities including the budget, there has been a potential problem that the outsourcing fees to the facilities and the amount of co-payment by the users vary widely. The issue is whether the same support and subsidies can be provided in the municipality where the mother returned home for delivery and does not have a certificate of residence. It will be considered necessary to establish a common system and procedures, such as a system in which the municipality where she has a certificate of residence compensates for the support.

## Mental healthcare at postpartum care facilities

Mental healthcare and/or childcare support have been provided to mothers at postpartum care facilities in collaboration with clinical psychologists, medical social workers, and medical staff depending on the scale of the institute.

The mental health statuses are often strongly related with the financial and social problems (5, 6). Therefore, by collaborating with multiple professions to solve and support these problems, the mental burden of postpartum women will be reduced, and they will be able to actively face their childcare. While it is impossible to solve and support financial and social problems through intervention by medical staff alone, it is also impossible to fully evaluate the necessary intervention without an approach from the medical staff. The importance will be to understand the severity of the mental status and to clarify the division of roles and methods of cooperation among the various professions. A community-wide attitude of looking after the mothers with problems will be required.

Nursing is the most important position in mental healthcare in obstetric institutes, and midwives in particular play the role of professionals who can provide the closest support to the mothers and their families (5, 6). Specifically, it is important to first listen to the feelings and words of all mothers and then empathize with their feelings and show empathy. By accepting the situation and supporting them, a relationship of trust will be built and become the basis for imaging solutions together with the mothers. Even if the mother is referred to another specialist in a variety of professions including psychiatry, the nursing staff should conduct a relationship of trust to avoid the misunderstanding that the mother has been abandoned and to ensure that she can continue to be consulted.

Once again, it is now important to accept the consultation content and feelings of mothers and show empathy under the Postpartum Care Program in Japan. At last, a recent report indicated that this program has been steadily expanding in Japan (7). On the other hand, an evaluation of whether or not the program is preventing adverse outcomes for mothers and infants remains to be conducted.

## References

[1] Ministry of Health, Labor and Welfare. Prenatal and Postnatal Support Business Guidelines/Postnatal Care Business Guidelines (in Japanese). (2020). Available online at: https://www.mhlw.go.jp/content/000658063.pdf (accessed November 5, 2023).

[2] Suzuki S. Current strategies for perinatal mental health care in Japan. JMA J. 7(1):5–9. 10.31662/jmaj.2023-0093 38314413 PMC10834275

[3] Suzuki S. National Subsidy Program for screening of postpartum depression in Japan. Asian J Psychiatr. (2022) 73:103151. 10.1016/j.ajp.2022.103151 35594687

[4] Suzuki S. Single center analysis of Japanese homecoming delivery and postpartum depression. JMA J. (2022); 5: 362–5. 10.31662/jmaj.2022-0069 35992290 PMC9358151

[5] Suzuki S. Appropriate support for “specified expectant mothers”. JMA J. (2022) 5: 17–22. 10.31662/jmaj.2021-0165 35224256 PMC8824720

[6] Suzuki S Eto M. Current status of social problems during pregnancy at a perinatal center in Japan. JMA J. (2020) 3: 307–12. 10.31662/jmaj.2020-0056 33225102 PMC7676988

[7] Hoshi SI Suzuki S Sagara Y Sekizawa A Ishiwata I. Expansion of mental health care in Japanese obstetric institutes. Cureus. (2024) 16: e54637. 10.7759/cureus.54637 38405651 PMC10884786

